# Clinical Trial: The Effects of Emulsifiers in the Food Supply on Disease Activity in Crohn's Disease: An Exploratory Double‐Blinded Randomised Feeding Trial

**DOI:** 10.1111/apt.70041

**Published:** 2025-02-18

**Authors:** Jessica A. Fitzpatrick, Peter R. Gibson, Kirstin M. Taylor, Ellen J. Anderson, Antony B. Friedman, Zaid S. Ardalan, Rebecca L. Smith, Emma P. Halmos

**Affiliations:** ^1^ Department of Gastroenterology School of Translational Medicine, Monash University and Alfred Health Melbourne Victoria Australia

**Keywords:** food additive, inflammation, inflammatory bowel disease

## Abstract

**Background:**

Advice to avoid dietary emulsifiers in Crohn's disease (CD) is based on preclinical data.

**Aims:**

To examine the effect of diets high (HED) and low (LED) in emulsifiers in the food supply on disease activity in CD.

**Methods:**

In a double‐blinded, randomised feeding study, we randomised adults with symptomatic, sonographically active CD with ileal involvement on ≥ 2 months' stable medical therapy to 4 weeks of a HED or LED modelled on Australian healthy eating guidelines. We measured the Harvey‐Bradshaw Index (HBI), sonographic indices (IBUS‐SAS, bowel wall thickness), quality of life (QOL) and fatigue at baseline and study completion.

**Results:**

We randomised 24 patients, mean age 37 (95% CI 32, 41) years, 12 male, HBI 6 (6, 8), bowel wall thickness 6.0 (5.5–6.6) mm. Adherence was > 95%. Clinical remission (HBI < 5) occurred in 9/12 on HED and 7/12 on LED; 2 and 3, respectively, withdrew early with increasing gastrointestinal symptoms. IBUS‐SAS fell from 51 (35, 68) to 33 (15, 51) on HED (*p* = 0.014) and from 57 (38, 76) to 44 (29, 59) on LED (*p* = 0.01). Bowel wall thickness reduced by 34% on HED and 15% on LED in those who completed the study. QOL and fatigue improved on both diets (*p* ≤ 0.05). There were no statistically significant differences in outcomes between diets.

**Conclusions:**

In the context of a healthy diet, the emulsifier content had no influence over disease activity over 4 weeks in patients with CD. Recommendations to avoid emulsifiers in patients with active CD are not supported.

Australian New Zealand Clinical Trials Registry (ACTRN12619001099112).

## Graphical abstract

Diets modelled on healthy eating guidelines, but differing in the content of emulsifiers available in the food supply, improved disease activity on sonographic criteria, Harvey‐Bradshaw Index and patient‐reported outcomes over 4 weeks in 24 patients with active Crohn's disease, with no differences according to the content of emulsifiers.

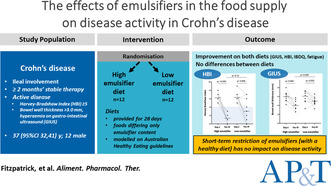

## 1 Introduction

Some dietary emulsifiers have been implicated in the pathogenesis of Crohn's disease from the findings of several preclinical studies. Exposure of specific emulsifiers or thickeners, mostly polysorbate‐80, carrageenan and/or carboxymethyl cellulose (CMC), to cell lines or Peyer's patches in vitro, bacteria in batch culture, mice susceptible to the development of intestinal inflammation and healthy humans has been associated with reduced barrier function, changes in behaviour and community structure of gut microbiota and, in mice, the development of intestinal inflammation [1, 2, 3, 4, 5, 6]. This is supported by prospective population studies that have shown an association between the intake of ultra‐processed foods (UPF) [7, 8, 9, 10], which commonly contain additive emulsifiers, and the development of Crohn's disease. Despite the lack of human clinical trial data, the message of emulsifiers causing or worsening intestinal inflammation is gathering momentum, with The European Society of Parenteral and Enteral Nutrition [11] and the International Organisation for the Study of Inflammatory Bowel Disease [12] recommending restriction or exclusion of UPF and specifically dietary emulsifiers in patients with Crohn's disease.

Yet, there are several concerns regarding translating experimental preclinical data and epidemiological associations into clinical practice. First, the emulsifier, polysorbate‐80 and the thickener, CMC, are uncommon additives in the food supply [13, 14, 15]. In fact, there are over 50 different emulsifiers and thickeners in UPF [13], most of which have not been subject to relevant studies. Therefore, while preclinical data are hypothesis‐generating, their clinical significance in patients with IBD who are very unlikely to be consuming these emulsifiers regularly is dubious. Second, the dosages used in preclinical studies have generally been supra‐physiological. For example, polysorbate‐80 or CMC were applied at 1% concentration in the drinking water of mice, which is estimated to be more than 500‐fold the anticipated exposure in humans consuming a diet high in emulsifiers [13]. Third, single emulsifiers have been tested in pure form, outside of the context of a complex food matrix and not in association with other emulsifiers.

Furthermore, intestinal barrier function improved, rather than worsened, with three‐weeks' exposure to a high‐emulsifier diet in healthy humans compared with that with a low‐emulsifier diet or the participants' own habitual diet [16]. There was also no discernible effect on inflammatory indices. However, high‐emulsifier intake appeared to sensitise the intestinal barrier to the detrimental effect of acute stress, and low emulsifier intake seemed to protect from this effect [16]. How these effects relate to patients with established Crohn's disease requires investigation.

In view of such uncertainties, an exploratory study was performed, which aimed to examine the effects of a low‐emulsifier diet that would comply with the current recommendations on restricting emulsifiers compared with those of a high‐emulsifier diet on disease activity and quality of life in patients with active Crohn's disease with ileal involvement. Hence, a double‐blinded, randomised, parallel‐group feeding study was undertaken in which the effects of a high‐emulsifier diet (HED) and a low‐emulsifier diet (LED) modelled on Australian healthy eating guidelines were assessed for 4 weeks. Disease activity was evaluated objectively by intestinal ultrasound and biochemical markers, more subjectively by a standard clinical disease activity index and by patient‐reported outcomes including quality of life and specific symptoms. A protocol in which nearly all food was provided was undertaken to maximise dietary adherence.

## 2 Methods

### 2.1 Participants

Between September 2022 and March 2024, patients with Crohn's disease were recruited from public hospitals and private practice consulting rooms across Melbourne, Australia. Inclusion criteria were a diagnosis of Crohn's disease with ileal involvement; the presence of clinically active disease defined by the Harvey Bradshaw Index (HBI) between 5 and 16 [17]; evidence of mild to moderate active inflammation on cross‐sectional imaging of the intestine with gastrointestinal ultrasound (GIUS); on stable medical therapy for at least 2 months; and aged between 18 and 60 years of age. Exclusion criteria were exposure to corticosteroids within the previous 4 weeks; the presence of a stoma, subtotal or total colectomy; current use of restrictive diets (including vegetarian/vegan); exposure to antibiotics, any supplementary types of probiotics or prebiotics within the previous 2 weeks or other therapies known to affect gastrointestinal function, including nonsteroidal anti‐inflammatory drugs; extremes of body mass index (< 18.5 or > 35 kg/m^2^); pregnancy/breastfeeding; significant mental health illness; and inability to give informed consent.

For eligible participants, current Crohn's disease medical therapy, disease location, phenotype, disease duration and resection history were obtained from medical records and/or their attending physician. Smoking status was self‐reported by participants.

### 2.2 Study Design

A randomised double‐blinded, parallel‐group, feeding study was performed. During a baseline period, participants completed a 7‐day habitual food diary using the EasyDietDiary mobile application on their smartphone [18]. They entered their dietary intake using household serves and weighed food measurements, including details about ingredients, brands of food and cooking methods. Participants recorded their gastrointestinal symptoms daily via an electronic REDcap survey. Eligible participants were then randomised in a one‐by‐one factor design (without blocking) using http://www.randomization.com to receive 28 days of a HED or LED, similar to those previously studied in healthy subjects [16]. All food was provided for both diets, apart from daily fresh fruit and salad ingredients. The research assistant (gastroenterology dietitian) who performed the randomisation and who responded to participant queries regarding dietary matters was not blinded but was not otherwise involved in data collection or analysis. Participants, the study coordinators, laboratory staff and gastroenterologists performing GIUS were blinded to the interventional diet the participants were receiving. To assist with participant blinding, the consent documents described the dietary interventions as exploring new therapeutic diets to treat Crohn's disease, but the words ‘emulsifiers’ or ‘food additives’ were not used.

Study visits were conducted in the morning with participants attending in a fasted state. At each visit, the participants were asked about adverse events and were encouraged to call the study coordinator or to enter them into their daily diaries. Height was measured at the first appointment. Weight was measured by the study coordinator at each study appointment, with participants removing their shoes and in light clothing only (SECA scale model mBCA 555, Hamburg, Germany). Waist circumference was measured around the abdomen while the participant was standing, at the midpoint between the lowest rib and top of the pelvic bone by the study coordinator at each study appointment. Body mass index was calculated. At their two study visits, day 0 and 28, participants brought with them a freshly passed faecal sample in a container that was immediately stored in a freezer at home and removed for transportation to the clinic appointment, where it was returned to a −20°C freezer and then stored at −80°C in the laboratory. Peripheral venous blood samples were collected from which serum was extracted and stored at −80°C in aliquots.

The study protocol was approved by Alfred Health Human Ethics Committee study ID number: 394/19 and registered at the Australian New Zealand Clinical Trials Registry (ACTRN12619001099112). All authors had access to the study data and reviewed and approved the final manuscript.

### 2.3 Interventional Diets

The HED and LED had been developed and evaluated for palatability, tolerability and blinding in 10 healthy subjects for one‐week each [13] as described elsewhere. Briefly, the HED and LED comprised foods purchased from Australian supermarkets differing only in the presence or absence of dietary emulsifiers/thickeners and were designed to meet Australian Healthy Eating Guidelines, which do not specify UPF or emulsifier intake [19]. The HED and LED were matched in energy, all macronutrients, micronutrients, fibre and fermentable oligo‐, di‐ and mono‐saccharides and polyols (FODMAPs). The diets were also matched for food groups, with both diets providing two serves of fruit, five serves of vegetables, three serves of dairy, one to two serves of meat/meat alternatives and four to six serves of grains per day. Legumes were provided three times a week and nuts were provided twice a week. Discretionary items (typically classified as junk food) were provided twice as snacks and twice as desserts per week. Meal plans for the HED and LED diets are described in Figure S1. Emulsifiers in the HED were in every food that was packaged, for example, bread, cereals, yoghurts, pre‐made sauces, biscuits, dips, cream cheese, gluten‐free pasta, microwavable rice, soups, nut spreads and pre‐made frozen meals. They were not present in the frozen vegetables. For packaged ingredients or food products, emulsifiers were deemed to be present based on the labelled ingredients listing the emulsifiers/thickeners. These were identified by CODEX e numbers (322, 400–499, 1100–1400). In brief, the HED contained 41 different emulsifiers. Of packaged products, 54% were able to be quantified for emulsifiers by weight (mg/per day), providing 2.8 g/day of added emulsifiers. Acetylated distarch adipate, whey powder, hydroxypropyl distarch phosphate, maltodextrin and mono‐diglycerides by weight were the most prevalent emulsifiers. Of emulsifiers of interest, polysorbate 80 was provided at 0.000024 mg/day and carrageenan at 4.4 mg/day. Food composition of CMC was not available, but it was known to be present in two products over the week [13]. Meat and dairy that naturally contain emulsifiers were permitted on both diets to observe Australian Healthy Eating Guidelines without inclusion of additive emulsifiers from meat and dairy alternatives. However, eggs (also containing natural emulsifiers) were excluded from the LED.

Participants were provided 4 weeks of each diet, comprising three main meals and three snacks daily. Meals and dry goods were prepared by the research chef, student dietitians and the study coordinator in accordance with food safety guidelines. They were portioned, vacuum‐sealed and then frozen. All dry consumables were taken out of the packet, portioned and vacuum‐sealed. The food was labelled with instructions on how to reheat or cook the meal as appropriate, provided free of charge and was delivered to the participants at the start of the diet and halfway through the study. Participants were instructed to eat to their appetite. Additional lists of foods not containing emulsifiers were provided to participants so they could purchase fresh fruit and vegetables if they were still hungry. If participants intended to eat meals outside of their home or wanted to include foods that were not specified on the supplied lists, they contacted an unblinded research assistant for guidance about what foods would be suitable. Foods not containing added emulsifiers were advised for each diet. Alcohol was not permitted on HED or LED.

### 2.4 Dietary Assessment

Participants recorded their intake of food daily in a food diary. This enabled the nutritional compositions of their habitual and interventional diets to be analysed with FoodWorks (Xyris software version 10, Brisbane, Australia) and by application of the NOVA criteria for UPF [20]. FODMAP intake was assessed using the Monash University database. The content of FODMAP oligosaccharides (fructo‐ and galacto‐oligosaccharides) and polyols (mannitol and sorbitol) was used as the most acceptable surrogate measure of total FODMAP intake [21]. Analysis of the food intake also permitted analysis of adherence to the provided diets, with adherence being defined as consuming only the food provided > 95% of the time. Food diaries were checked by the research assistant (gastroenterology dietitian) upon their return.

### 2.5 Outcome Measures

Since this was an exploratory study, all outcome measures were considered exploratory, with a view to generating hypotheses for further study. These measures covered the assessment of disease activity including GIUS and faecal calprotectin, specific symptoms and quality of life.

#### 2.5.1 Measures of Disease Activity

Disease activity was assessed with three measures at each study visit:

##### 2.5.1.1 Clinical Disease Activity

The HBI was calculated by the study coordinator prior to and at the end of the dietary intervention.

##### 2.5.1.2 Gastrointestinal Ultrasound

GIUS was performed by gastroenterologists from the Alfred Hospital IBD Service. They were accredited for this according to criteria defined by the GastroEnterology Network for Intestinal Ultrasound (GENIUS) and the International Bowel Ultrasound Group (IBUS). All had performed at least 2500 examinations at the time of study commencement. They were blinded to intervention diets.

The protocol for GIUS has been described in detail elsewhere [22, 23]. Examinations were performed using a Canon Aplio i800 machine (Canon Medical Systems Corporation, Otawara‐shi, Tochigi, Japan) with no preparation prior to examination. Examination commenced with a convex 1–8 MHz transducer followed by a linear 3–11 MHz transducer, with the linear probe being used to assess the bowel segments. Each gastroenterologist was provided with the same record sheet to assess every segment of the bowel in its entirety, regardless of disease distribution, to ensure a complete examination. The sigmoid, descending, transverse and ascending colon, the caecum, terminal ileum (or neo‐terminal ileum), anastomosis and small bowel segments were examined. Bowel wall thickness (BWT) was recorded in mm for the most affected segment. Hyperaemia (vascularity) of the bowel segments was assessed by Doppler and recorded as active, mild, moderate or severe using a modified Limberg score (grade 0 = absent, grade 1 = short signals, grade 2 = long signals inside bowel, grade 3 = long signals inside and outside bowel) [24]. Wall stratification, mesenteric hyperechogenicity and lymphadenopathy were also recorded.

Sonographic response was defined as a reduction in BWT by ≥ 2 mm or a reduction in BWT by < 1 mm and a reduction in hyperaemia or a 25% reduction in BWT [25]. The International Bowel Ultrasound Segmental Activity Score (IBUS‐SAS) was calculated for the most affected segment by assessing the most affected bowel segment, as described elsewhere [24].

##### 2.5.1.3 Biochemical Measures

These were evaluated prior to and at the end of the dietary interventions. Serum concentrations of highly sensitive C‐reactive protein (CRP) were measured by nephelometry by the on‐site pathology service. The normal range was < 4 mg/L. Faecal calprotectin was measured by Calpro Easy Extract (Svar, Lysaker Norway) as a single batch by ELISA (Elitech Group, Braeside, Australia) as per the manufacturer's instructions and expressed as μg/g faeces. Response was arbitrarily defined as a fall of calprotectin by 25%.

#### 2.5.2 Symptoms

Symptoms, comprising overall gastrointestinal symptoms, abdominal pain and fatigue, were scored daily on a 100‐mm visual analogue scale (0 indicating no symptoms and 100 indicating worse possible symptoms) [26, 27]. A Redcaps survey was sent to participants in a text message daily. Average weekly scores were calculated.

#### 2.5.3 Quality of Life

Quality of life was measured before and at the final study appointment at the end of the dietary interventional period using the inflammatory bowel disease questionnaire (IBD‐Q) [28]. Using the scoring metric, individual scores for the four domains, bowel system, emotional health, systemic systems and social function, were generated as well as an overall IBDQ score. A clinically meaningful improvement in quality of life was defined as an increase of ≥ 16 points in the IBDQ total score.

### 2.6 Statistical Analysis

Since there were no precedent data on the likely effects of whole food‐based diets on objective disease activity in adult patients with active Crohn's disease, the number of patients to be studied was judged on the basis of the feasibility of performing a feeding study with a duration of 4 weeks for the intervention, together with our experience of the previous pilot evaluation of similar diets in healthy humans [29]. Twenty‐four participants were considered to be the minimum for such a study.

Descriptive statistics were presented as mean and 95% confidence intervals, or median and interquartile range (IQR), depending on data distribution. Differences between paired data were analysed by paired *t*‐test or Wilcoxon signed rank test, and between non‐paired data were analysed by Student's *t*‐test or Mann–Whitney test or Kruskal–Wallis test, depending on the normality of the data and number of groups. Proportions were compared using Chi‐square test or Fisher's exact test. Time course of symptoms was analysed via linear regression analysis. Data were analysed both as intention‐to‐treat (ITT) and per‐protocol (PP), as indicated. Apart from where Bonferroni corrections were made for multiple comparisons, a *p*‐value ≤ 0.05 was considered statistically significant. Prism software (version 9.5.1 (528); GraphPad Software, San Diego, CA) was used for analysis.

## 3 Results

### 3.1 Participants

**FIGURE 1:**
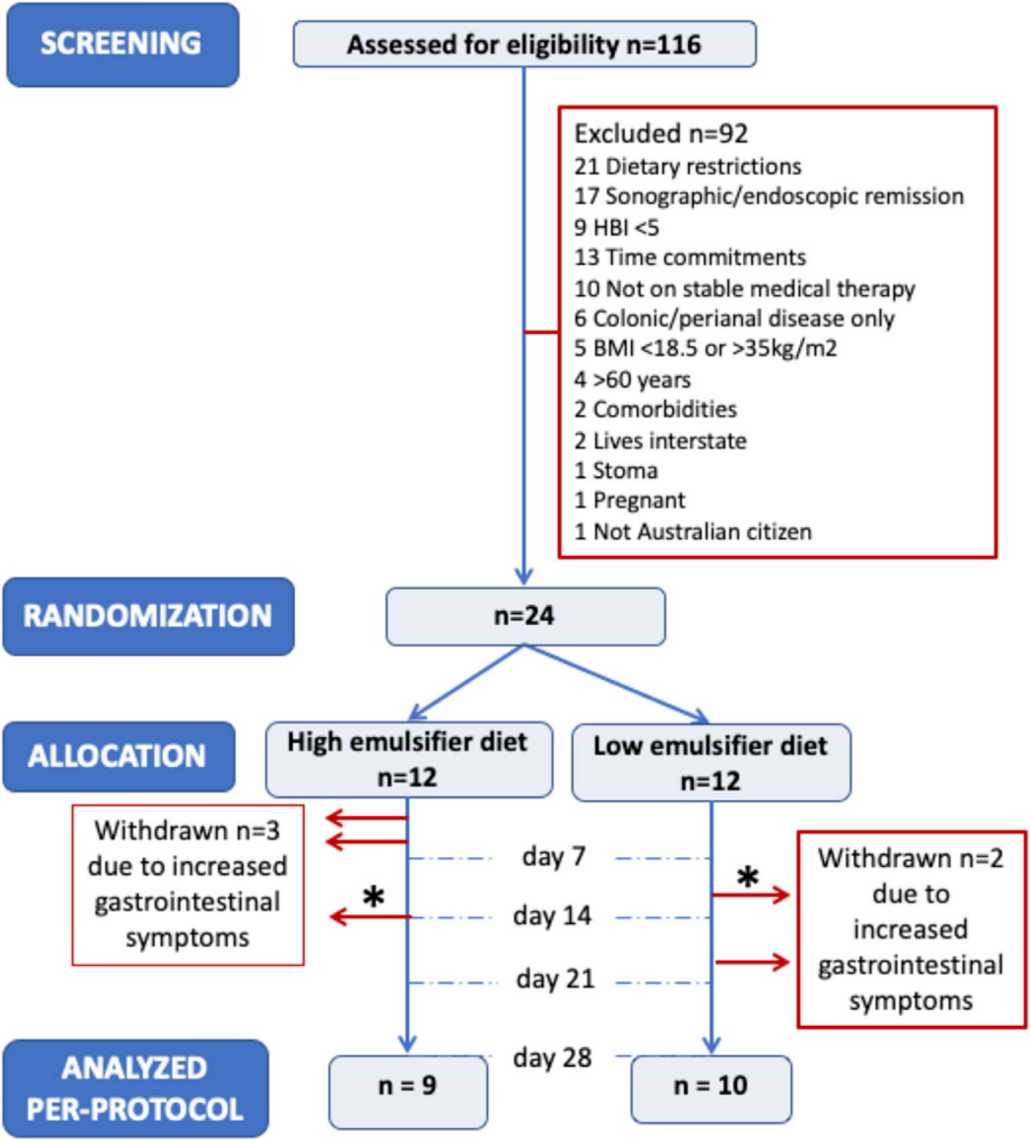
CONSORT diagram. * Indicates that gastrointestinal ultrasound was performed as patient withdrawal.

Of 116 patients screened for eligibility, 24, mean age 37 (95% CI 32, 41) years and 12 male, were enrolled and randomised (Figure 1). Three patients withdrew from the HED and 2 from the LED due to increased gastrointestinal symptoms. One patient from each dietary group had an exit GIUS (Figure 1). Patient demographics and clinical details were similar between the two randomised groups (Table 1).

**TABLE 1:** Baseline patient demographics and clinical features according to the dietary intervention.

	Diet		*p*
	High emulsifier	Low emulsifier	
Number of patients	12	12	
Sex: male, *n* (%)	6 (50)	6 (50)	> 0.99
Age, years	36 (29, 43)	35 (29, 41)	0.89
Body mass index (kg/m^2^)	25.2 (22.8, 27.5)	25.9 (23.6, 28.2)	0.65
Ethnicity, *n* (%)			
White	12 (100)	11 (92)	> 0.99
Asian	0	1 (8)	
Smoking status, *n* (%)			
Current smoker	0	0	0.37
Ex‐smoker	5 (42)	2 (17)	
Never smoker	7 (58)	10 (83)	
Harvey Bradshaw index	6 (5,7)	7 (6,7)	0.30
Serum C‐reactive protein, mg/L^a^	2.2 (0.7–6.2)	2.6 (0.7–4.3)	0.92
Faecal calprotectin, μg/g^a^	108 (66–180)	102 (43–260)	0.76
Disease duration, years	7.2 (2.1, 12.3)	14.7 (7.0, 22.5)	0.086
Age at diagnosis, *n* (%)			
A1 < 16 years	1 (8)	3 (25)	0.20
A2 17–40 years	8 (67)	9 (75)	
A3 > 40 years	3 (25)	0	
Disease location, *n* (%)			
L1 – terminal ileum	10 (83)	5 (42)	0.084
L2 – colon	0 (0)	0 (0)	
L3 – ileocolonic	2 (17)	7 (58)^b^	
Disease behaviour			
B1 – inflammatory	7 (58)	4 (33)	0.41
B2 – stricturing	3 (25)	6 (50)	
B3 – penetrating	2 (17)	2 (17)	
P – perianal	(1)	0 (0)	
IBD related surgery (*n*, %)	4 (33)	4 (33)	> 0.99
Steroid use in past 3 months	0 (0)	2 (17)	0.48
Medical therapy			
No therapy	6	5	0.86
Immunomodulators	5	4	
Mesalamine	0	1	
Advanced therapies^c^	4	6	
Stable dose duration, years	2.4 (0, 4.9)	0.8 (0.3, 1.3)	0.086
Escalated dosing, *n* (%)	2 (17)	3 (25)	> 0.99
Inflammatory bowel disease questionnaire	158 (140, 177)	143 (127, 158)	0.17
Symptoms on visual analogue scale, mm			
Overall GI	35 (22, 47)	43 (35, 52)	0.22
Abdominal pain	32 (20, 45)	40 (28, 53)	0.33
Fatigue	52 (38, 67)	57 (50, 65)	0.57

*Note:* Data presented as mean (95% CI), unless specified; analysed by Student's *t*‐test or Fisher's exact test or chi‐square test.

^a^ Data presented as median (IQR) and compared via the Mann–Whitney *U* test.

^b^ *n* = 1 with previous upper gastrointestinal involvement.

^c^ Adalimumab *n* = 4, infliximab *n* = 4, ustekinumab *n* = 1, vedolizumab *n* = 1.

### 3.2 Food Intake

Food composition analysis of food diaries completed on the habitual and provided diets is presented in Table 2. There were no differences in macronutrients or their contribution to energy per day between the habitual and interventional diets. Fibre, total FODMAP content [driven by higher fructo‐oligosaccharides (FOS)] was higher in the intervention diets compared to habitual diets, with differences in UPF content across the diets. Sugar content was higher in HED and LED compared with habitual diet intake. Alcohol content range during habitual diet was 0–150 g.

**TABLE 2:** Nutritional composition of habitual and interventional diets.

Nutrient	Units	Habitual diet	Interventional diets		*p*	
			High emulsifier	Low emulsifier	High vs low‐emulsifier diet^a^	Across the 3 diets^b^
Energy	MJ/d	7.9 (6.3–10.7)	8.4 (7.7–9.7)	8.5 (7.8–10.0)	0.84	0.66
Protein	g/d	95 (73–120)	85 (64–99)	89 (70–111)	0.38	0.38
	% Energy	19 (15–22)	15 (12–19)	15 (14–21)	0.38	0.10
*Fat*						
Total	g/d	72 (50–102)	70 (62–106)	74 (62–99)	0.90	0.94
	% Energy	34 (29–40)	34 (28–37)	32 (26–40)	> 0.99	0.88
Saturated	g/d	29 (21–40)	31 (29–38)	29 (20–43)	0.43	0.81
	% Energy	13 (9–16)	13 (12–14)	11 (10–16)	0.46	0.69
*Carbohydrate*						
Total	g/d	201 (158–266)	244 (195–281)	235 (202–267)	0.929	0.24
	% Energy	43 (37–49)	49 (43–56)	43 (40–52)	0.74	0.19
Sugar	g/d	61 (43–87)†*	103 (90–188)†	88 (73–93)*	0.031	0.002
	% Energy	13 (9–18)†	21 (17–25)†	17 (15–19)	0.053	0.003
Fibre	g/d	19 (14–25)†*	29 (23–36)†	35 (27–37)*	0.34	< 0.001
*FODMAPs (g)*						
Total^c^	g/d	4.0 (2.9–5.5)†*	6.1 (4.3–9.9)†	5.7 (4.7–9.9)*	0.90	< 0.001
FOS	g/d	2.4 (1.8–1.3)†*	4.4 (3.4–5.1)†	3.8 (3.4–4.9)*	> 0.99	0.003
GOS	g/d	0.5 (0.3–3.3)	0.6 (0.4–1.0)	0.7 (0.5–0.9)	0.74	0.78
Sorbitol	g/d	0.3 (0.2–0.8)	1.1 (0.1–4.9)	0.9 (0.0–4.7)	0.48	0.33
Mannitol	g/d	0.3 (0.2–0.6)	0.5 (0.0–1.6)	0.5 (0.0–1.6)	0.79	0.93
Ultra‐processed foods^d^	MJ/d	3.5 (1.9–6.0)†	6.3 (5.4–6.8)†	2.3 (1.9–3.0)	< 0.001	0.014
	% Energy	46 (26–63)†*	68 (63–82)†	27 (22–34)*	< 0.001	0.002

*Note:* All data presented as median (IQR). Food diaries available for *n* = 11/12 HED and 11/12 LED.

Abbreviations: FODMAP, fermentable oligosaccharides, disaccharides, monosaccharides and polyols; FOS, fructo‐oligosaccharides; GOS, galacto‐oligosaccharides; HED, high‐emulsifier diet; LED, low‐emulsifier diet.

^a^ Compared using the Mann–Whitney test.

^b^ Kruskal–Wallis test, *p*‐value < 0.007 was considered statistically significant after Bonferroni correction for multiple comparisons.

^c^ Total FODMAP oligosaccharide and polyol intake used as a surrogate marker of total FODMAP intake.

^d^ Level 4 by NOVA classification.

*Note:* † *Significantly different compared using the Mann–Whitney test.

### 3.3 Dietary Adherence

All participants adhered completely to the provided diets, with two exceptions. One participant on the LED consumed foods likely containing emulsifiers (chocolate, blueberry muffin, sushi rolls) on 3 of the 28 days’ dietary intervention. One participant on the HED consumed one meal that was prohibited (risotto, baked eggplant and sorbet) with sorbet likely to contain emulsifiers.

### 3.4 Effect of Interventional Diets on Disease Activity

#### 3.4.1 Clinical Disease Activity Score

**FIGURE 2:**
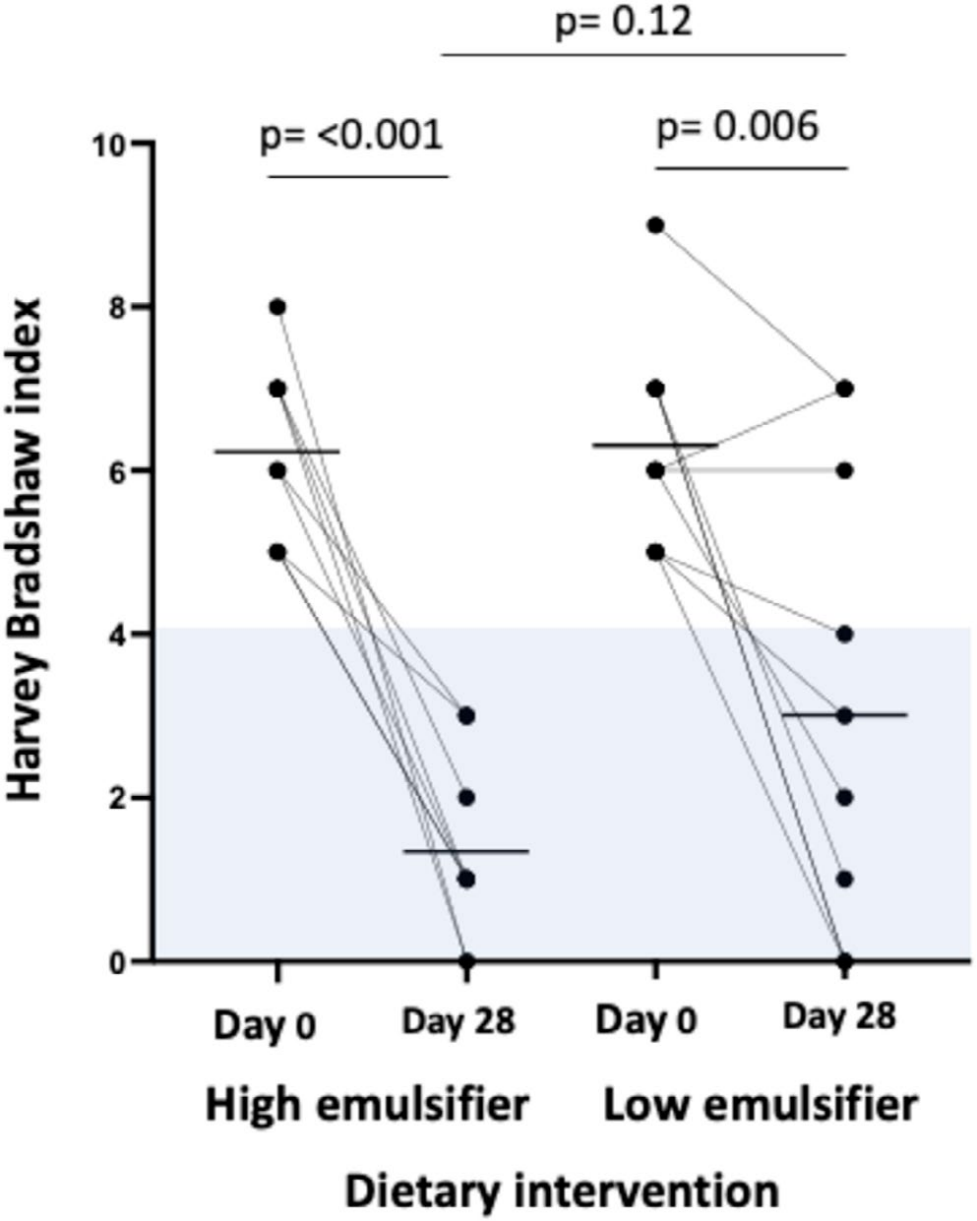
Harvey‐Bradshaw Index at day 0 and after 28 days on high and low emulsifier diets. Black lines indicate mean, blue shading indicates clinical remission (HBI < 5). Analysed via paired and non‐paired *t*‐test. Statistical significance *p* ≤ 0.05.

On ITT analysis, 9/12 on HED and 7/12 on LED were in clinical remission at the end of the dietary intervention, with no difference between the diets (*p* = 0.67). When analysed per‐protocol, all nine patients were in clinical remission on the HED (HBI median 1 [IQR, 0–2]) and 7/10 patients were in clinical remission on the LED (HBI 3 [1–5]), with no difference between the diets (*p* = 0.21) (Figure 2). The participant who did not adhere to the LED reduced their HBI from 5 to 3.

#### 3.4.2 Gastrointestinal Ultrasound

**FIGURE 3:**
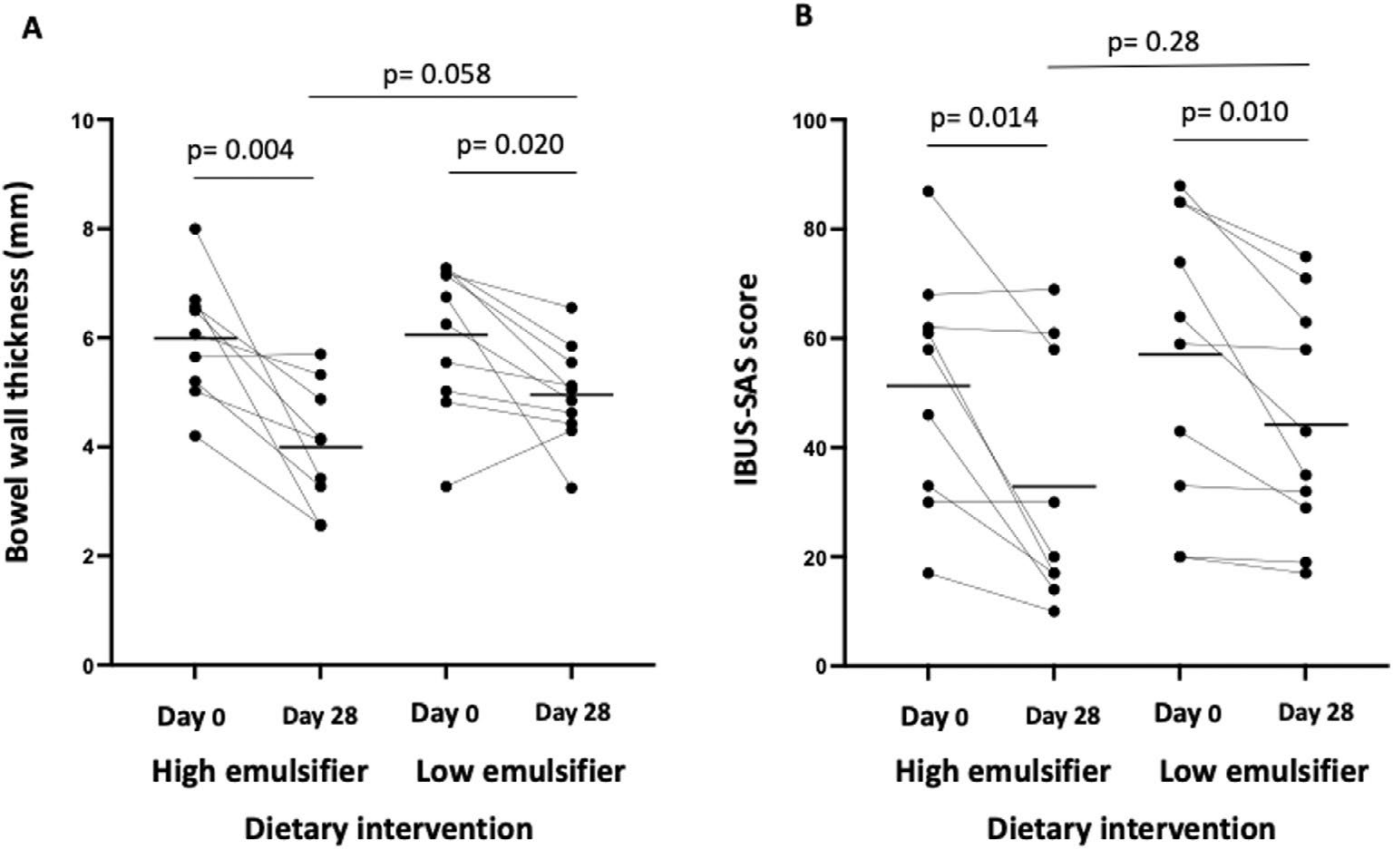
(A) Bowel wall thickness and (B) International Bowel Ultrasound Segmental Activity Score (IBUS‐SAS) on high and low‐emulsifier diets at day 0 and day 28 measured by gastrointestinal ultrasound. Black lines indicate mean. Analysed via paired and non‐paired *t*‐test. Statistical significance *p* ≤ 0.05.

In an ITT analysis, sonographic response was seen in 7/12 on HED and 4/12 in LED (*p* = 0.41). In the per‐protocol analysis, sonographic response was seen in 7/9 in HED and 4/10 in LED (*p* = 0.17) for those who completed the diets. Bowel wall thickness reduced significantly from that at baseline in both dietary groups by 34% (19%, 48%) with the HED and by 15% (0%, 30%) on LED (Figure 3A). The IBUS‐SAS fell from 51 (35, 68) to 33 (15, 51) on the HED (*p* = 0.014) and from 57 (38, 76) to 44 (29, 59) on the LED (*p* = 0.010) with no difference between the diets (Figure 3B). The participant who did not adhere to the LED was a non‐responder. Despite this, the BWT in this participant fell from 7.3 mm at baseline to 5.9 mm at the end of the dietary intervention, but hyperaemia remained moderate throughout the study.

#### 3.4.3 Biochemistry

**FIGURE 4:**
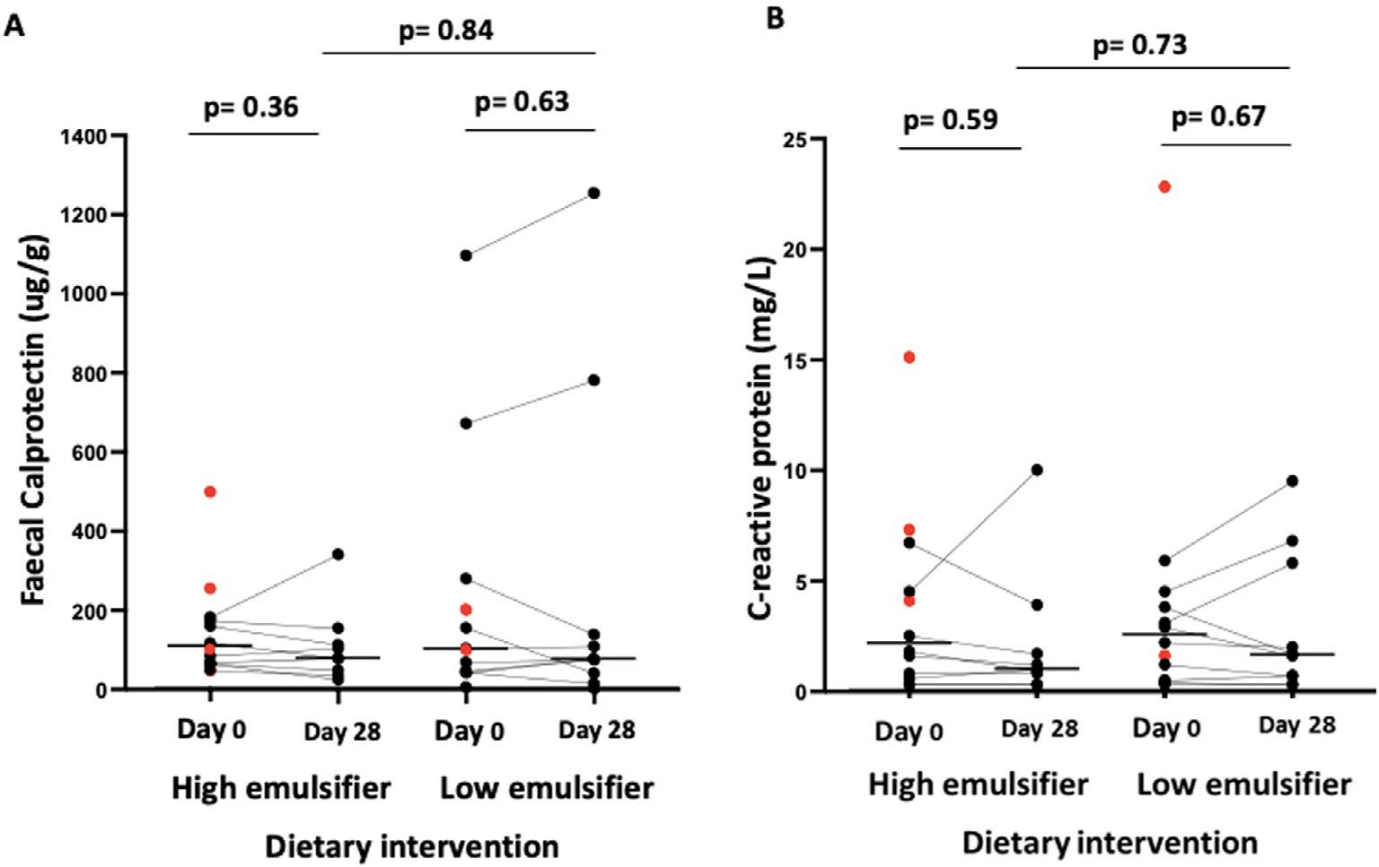
(A) Faecal calprotectin and (B) serum C‐reactive protein (CRP) on high and low‐emulsifier diets at day 0 and day 28. Solid black line indicating median, red dots indicating participants who withdrew. Analysed via Wilcoxon signed rank test or Mann–Whitney *U* test. Statistical significance *p* ≤ 0.05.

Nine of 19 participants (47%) had ≥ 25% reduction in faecal calprotectin, with 5 being in the HED and 4 in the LED (*p* = 0.66, per‐protocol). Overall, faecal calprotectin did not change after either dietary intervention, and there was no difference between diets (Figure 4A). The participant with a highly elevated faecal calprotectin at baseline (1096 μg/g) on LED had active colonic inflammation. Concentrations of CRP did not change overall with the dietary interventions (Figure 4B).

### 3.5 Effect of Interventional Diets on Symptoms

**FIGURE 5:**
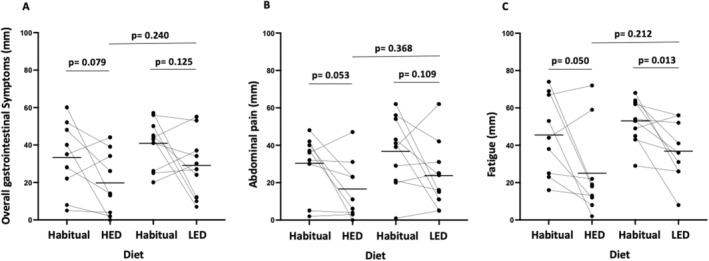
Effect of 28 days of high‐ (HED) and low‐ (LED) emulsifier diets on (A) overall gastrointestinal symptoms (B) abdominal pain and (C) fatigue, scored using a 100‐mm visual analogue scale (VAS). Black lines indicate mean. Analysed via paired and non‐paired t‐test. Statistical significance *p* ≤ 0.05.

**FIGURE 6:**
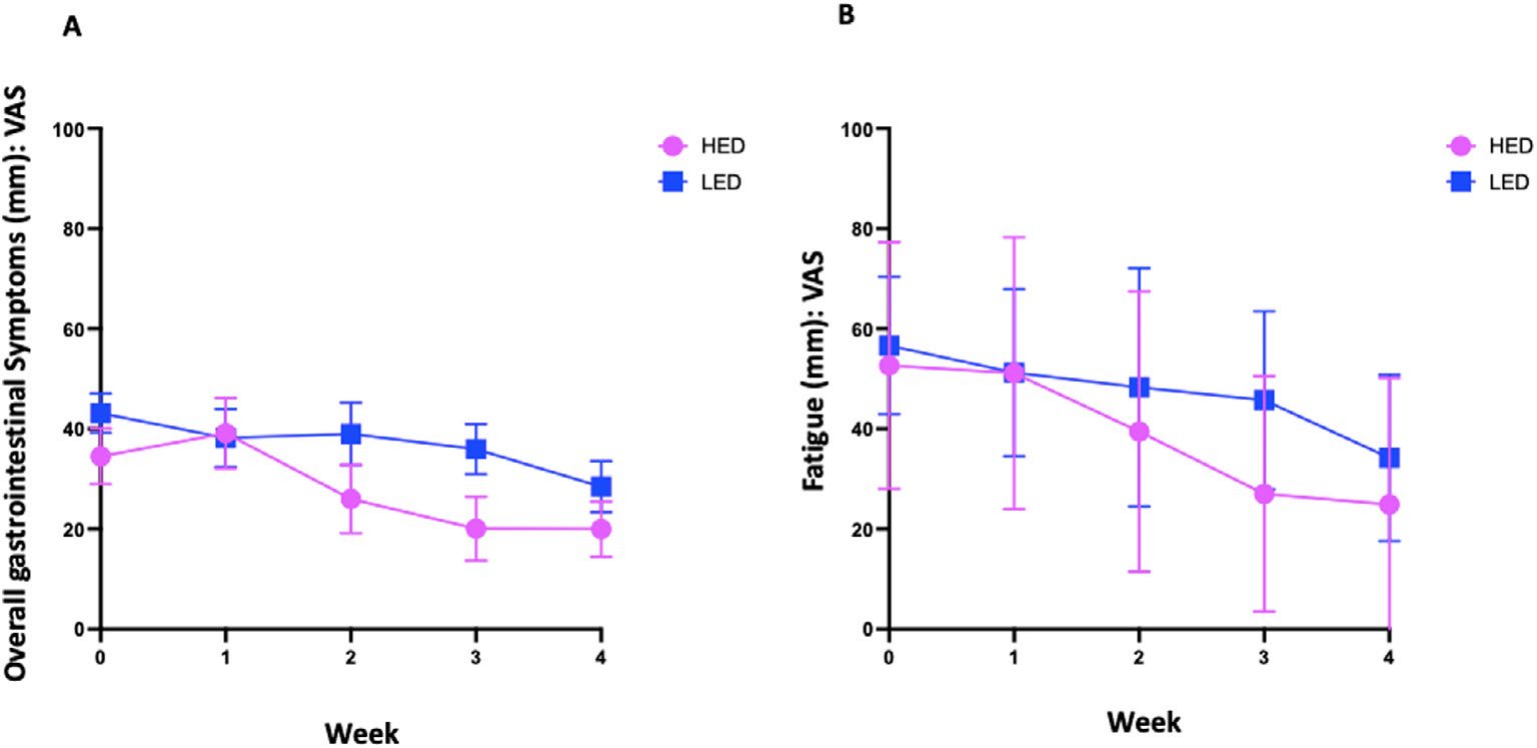
Weekly average scores for (A) overall gastrointestinal symptom and (B) fatigue on 100‐mm visual analogue scale (VAS) on high (HED) and low (LED) emulsifier diets.

There was a trend of improved overall gastrointestinal symptoms in the per‐protocol cohort on HED (habitual, 33 [18, 48] vs. end of study, 20 [6, 32]; *p* = 0.079) and the LED (41 [31, 50] vs. 29 [17, 41]; *p* = 0.125), with a similar trend for abdominal pain (Figure 5A,B). No differences were observed between the diets. Fatigue scores improved with both diets—HED (45 [29, 62] vs. 25 [7, 43]; *p* = 0.050) and LED (53 [44, 62] vs. 37 [26, 48]; *p* = 0.013), but there was no difference between the diets (Figure 5C). Improvements in weekly scores for overall gastrointestinal symptoms (Figure 6A) and fatigue (Figure 6B) occurred over the 4 weeks similarly for each diet.

### 3.6 Effect of Interventional Diets on Quality of Life

**FIGURE 7:**
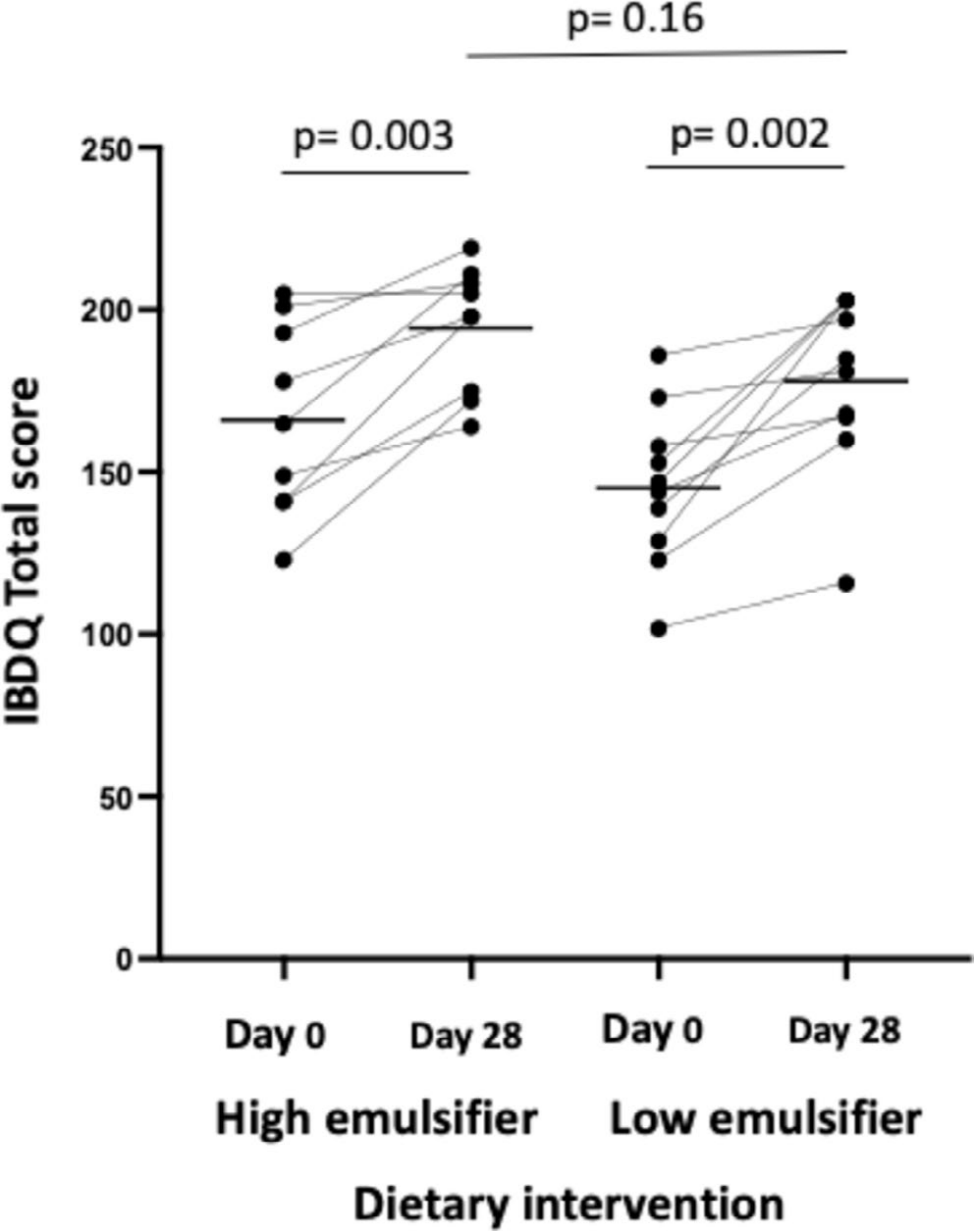
Quality of life response at day 0 and after 28 days on high and low‐emulsifier diets using the inflammatory bowel disease questionnaire. Black lines indicate mean. Analysed via paired and non‐paired *t*‐test. Statistical significance *p* ≤ 0.05.

Quality of life improved after 28 days on both diets, with the mean of both groups exceeding the clinically meaningful improvement, with no differences between the diets (Figure 7). All domains of the IBDQ (bowel, emotional health, systemic systems and social function) improved similarly on both diets (Table 3).

**TABLE 3:** Quality of life on high‐ and low‐emulsifier diets using the inflammatory bowel disease questionnaire (IBDQ). Data presented as mean (95% CI) and analysed by paired *t*‐test within a diet or unpaired *t*‐test between diets.

IBDQ domain	High‐emulsifier diet		*p*	Low‐emulsifier diet		*p*	*p* between diets at day 28
	Day 0	Day 28		Day 0	Day 28		
Bowel system	5.2 (4.6, 5.9)	5.9 (5.4, 6.5)	0.008	4.4 (3.7, 5.1)	5.7 (4.9, 6.4)	0.002	0.53
Emotional health	5.3 (4.3, 6.2)	6.2 (5.6, 6.8)	0.012	4.8 (4.2, 5.3)	5.7 (5.0, 6.3)	0.006	0.16
Systemic systems	4.4 (3.6, 5.2)	5.5 (4.9, 6.2)	0.019	3.6 (2.9, 4.3)	4.8 (4.0, 5.5)	0.006	0.11
Social functions	5.8 (3.6, 5.2)	6.6 (6.3, 7.0)	0.039	5.3 (4.5, 6.1)	6.4 (5.7, 7.0)	0.001	0.41
Total score	166.2 (143.5–189.0)	194.4 (179.5–209.3)	0.003	145.4 (128–162.8)	178.3 (158.8–197.8)	0.002	0.16

### 3.7 Effect of Interventional Diets on Anthropometric Measures

On the HED, participants lost mean of 2.21 (1.27, 3.16) kg from baseline (*p* < 0.001) and their waist circumference reduced from 90.2 (80.0, 100.8) cm to 87.5 (77.8, 97.2) cm (*p* = 0.007). On the LED, participants lost 2.48 (1.14, 3.82) kg from baseline (*p* = 0.002) and their waist circumference reduced from 88.8 (81.4, 96.1) cm to 84.5 (77.0, 92.0) cm (*p* = 0.006). There were no differences in the changes in weight (*p* = 0.73) or waist circumference between the diets (*p* = 0.30).

### 3.8 Adverse Events

Participants tolerated the diets well. Adverse events were gastrointestinal symptoms that increased in 5 (3 on HED and 2 on LED), leading to early withdrawal from the intervention (Figure 1). The CRP of patients withdrawing was 10.2 (0, 21.0) mg/L, which was higher than 2.3 (1.4, 3.3) mg/L in the patients who completed the study (*p* = 0.001). All of those who withdrew had a baseline faecal calprotectin ≥ 100 μg/g. Two patients underwent GIUS at withdrawal; at day 14, the GIUS findings were unchanged in the patient on the HED, while one patient on the LED withdrawing at day 11 had a new finding of colonic faecal loading, other findings being unchanged.

## 4 Discussion

Whether emulsifiers consumed in the food supply are detrimental to disease activity in patients with Crohn's disease has previously been unknown. This exploratory study compared the impact of 4 weeks of two dietary interventions modelled on Australian dietary guidelines [19], but differing only in the content of emulsifiers, in symptomatic adults with Crohn's disease with ileal involvement, in whom active inflammatory disease was present. We demonstrated that both diets were associated with improved clinical disease activity and sonographic evidence of intestinal inflammation, although convincing effects on faecal calprotectin were not observed. Furthermore, health‐related quality of life improved similarly with both diets, as did fatigue. These findings are contrary to popular beliefs and advice that emulsifiers are harmful. They showed no evidence of injurious effects of emulsifiers in the setting of an otherwise healthy diet as predicted by several reviews of preclinical and other experimental studies [30, 31, 32, 33, 34].

Two factors essential to the validity of a dietary intervention study are the certainty of adherence to the prescribed diet and the actual nutritional composition of experimental diets. The 7‐day meal plans were developed on the basis of what is eaten in the community from supermarkets and ensured the principles of healthy eating were followed. The diets were well tolerated and, as found in the lead‐up studies in healthy adults, were eaten without complaint [13, 16]. This has also been observed in the United Kingdom and Hong Kong [14, 35]. Detailed analysis of the diets consumed enabled confidence that nutrient content was similar and that no differences in components that potentially might affect symptoms, such as the FODMAP and fibre contents, were likewise similar. The only major differences were in the content of emulsifiers and of UPFs, by virtue of the fact that emulsifiers are integral to the processing of foods. One of the difficulties in quantifying dietary emulsifier content is the lack of available analytical methodologies. The alternative is to add up the amounts claimed to be present in the foods from the manufacturers. However, strict food labelling laws legislate the requirement to state the presence of emulsifiers in ingredient lists, but not the amount in foods, although there are safe limits defined by government authorities. We were able to obtain such information for 54% of the foods included; food manufacturers were unwilling to provide quantitative information on the other 46% [13]. With these limitations, participants on the HED were exposed to 41 different emulsifiers during the course of the week in the context of different foods, and multiple emulsifiers were consumed within one meal [13]. The HED provided at least 2.8 g/day of total emulsifiers a day, based upon the quantifiable foods used [13].

The importance of such an analysis of the intervention diets was to gain a perspective of overall and specific emulsifier intake in relation to those utilised in murine and human studies. The choice of emulsifiers in experimental studies has predominately been polysorbate 80, CMC and carrageenan. None of these are widely present in the Australian food supply; an audit of Australian packaged food revealed these emulsifiers present in 3, 60 and 138 products of 1680 products, respectively [13]. This has also been observed in the United Kingdom and Hong Kong [15, 35]. Accordingly, in the diets for the present study, these specific emulsifiers were found in very few foods. Even without precise quantification, the doses used in preclinical models are in gross excess of dietary human exposure. Examples include nearly 3‐million‐fold the dose of polysorbate 80 given to mice (after adjustment for size differences) and 583‐fold greater CMC in a human study [13]. Hence, the dietary intervention used in the current study reflected the worst‐case scenario in everyday real‐world exposure that IBD patients would be experiencing rather than pharmacological doses.

The patients for this study were carefully selected on the basis of phenotype (history of ileal involvement) essential for reducing phenotypic heterogeneity [36] and for suitability of accurate sonographic assessment, presence of sonographically active intestinal inflammation, clinical disease activity according to the HBI and stability of medication (to ensure any clinical change could not be ascribed to slow onset or offset of drug effects). Faecal calprotectin, which was measured as a single batch at the end of the study, provided limited insights; it was in the range often stated to be in remission (< 150 μg/g) in the majority of patients and was > 250 μg/g in only four patients at baseline. These observations highlight the sensitivity of GIUS in identifying intestinal inflammation and the weak correlation between BWT and calprotectin in patients with ileal disease. Previous evaluation of 105 patients with ileal Crohn's disease showed a sensitivity of 73%, and this was only when faecal calprotectin was over 100 μg/g [37].

No differences in outcomes were observed between the two diets, both diets being associated with improved outcomes. The time‐honoured measure of disease activity, the HBI, normalised in all patients consuming the HED to completion and in all but three with the LED. However, biochemical biomarkers were less informative since both the CRP and faecal calprotectin were within the normal range or minimally elevated in most patients. Reductions > 25% in faecal calprotectin were observed in about one half of the patients, but no change was observed overall. Of importance, both markers remained within the normal or generally acceptable limits in the majority of patients. In contrast, overall responses in sonographic activity in the per‐protocol cohort were consistent with a large effect size. GIUS is accurate with very small intra‐ and inter‐observer variance [38]. After 4 weeks of diet, the HED was associated with a 34% and 2.0 mm reduction of BWT and the LED with a 15% and 1.1 mm reduction in BWT. The magnitude of these changes compares favourably with those reported with biologic therapy. For example, reduction of BWT with 4 weeks of ustekinumab was 9.6% [39] and with 12 weeks of anti‐TNF therapy 1.1 mm [40]. This was associated with consistent improvement in the quality of life and fatigue scores. Overall gastrointestinal symptom and abdominal pain scores tended to fall from baseline, and this appeared to be a progressive fall to the 4 weeks.

Not all patients did well with the dietary interventions. Five (21%) withdrew from the study due to worsening of gastrointestinal symptoms, albeit 2 within the first week of the diet. The withdrawing patients had significantly greater CRP than those completing the diets. Only two had an exit GIUS, which showed similar scores for inflammatory activity, but one had developed faecal loading in the colon, a potential source for symptom exacerbation. The main signal from the withdrawing group was that patients with more severe inflammation may not be suitable for such dietary intervention. However, it should be noted that the rate of withdrawal was similar to 34% of 40 participants reported in the landmark study comparing adults who were randomised to receive the Crohn's Disease Exclusion Diet with partial enteral nutrition compared with the Crohn's disease diet alone [41].

Contributing factors to the clinical improvements observed deserve discussion since they are unlikely to relate to the content of emulsifiers or UPF. First, the improvements might have been placebo effects, but they were not restricted to patient‐reported outcomes. In meta‐analyses of randomised controlled trials, pooled estimates of the placebo rates of clinical remission were mean 18% (95% CI, 14%, 24%) [42] and of endoscopic response (in place of sonographic response for which there are no published data) were 13% (10%, 16%) [43].

Second, differences other than in the content of UPFs and emulsifiers require examination. It is likely that the quality of the habitual diet was poorer. Whilst the quality of the diets was not formally evaluated, the provided HED and LED did comply 100% with the Australian Guide to Healthy Eating while the average Australian adherence rate is 59% [44]. Fibre is often a proxy measure for a greater intake of high‐quality foods such as fruit, vegetables, legumes, wholegrains, nuts and seeds. Fibre content was higher in the interventional diets compared with that in the habitual diet, but the increment is unlikely to be the primary reason for an improvement in inflammatory activity. Contrary to popular opinion, a healthy diet based on national guidelines does not preclude or even necessarily minimise UPF or emulsifiers, as reflected in the 68% energy from UPF provided on the HED. UPF has a low threshold for definition, meaning many ‘healthy’ foods, such as supermarket bread, flavoured yoghurt and high‐fibre breakfast cereals would meet UPF classification. The impact of compliance with Australian guidelines for healthy eating has not been evaluated in reducing inflammation in active Crohn's disease. However, a 6‐month lifestyle intervention demonstrated better adherence to the national guidelines of the Netherlands and was associated with improvements in general well‐being and fatigue in a large cohort of quiescent IBD patients, although a large degree of dietary change was required to achieve these benefits [45].

Third, all patients lost weight during the 4‐weeks' interventions, despite the diets meeting energy requirements for the patients and being isocaloric for the habitual diet. This may reflect under‐reporting of intake on the habitual diet or, less likely, over‐reporting of intake on the interventional diets. This is a common challenge faced by dietary studies, with under‐reporting from food diaries being up to 41% of actual intake when compared to double‐labelled water energy expenditure techniques [46]. Adherence based on food diaries was high. The associated reduction in waist circumference implied the loss of central adiposity, which has been associated with intestinal inflammation through production of inflammatory cytokines such as TNF‐⍺ and IL‐6 from creeping fat seen in Crohn's disease [47]. Hence, the improved body composition of patients may have contributed to improved disease activity.

The findings in this study provide the first evaluation of the current advisory to avoid dietary emulsifiers in patients with Crohn's disease, based upon the potentially harmful effects of emulsifiers within the food supply on intestinal barrier function, mucus layer, inflammation and alterations to the gut microbiome that have excited much concern [15, 30, 31, 33, 48]. Such advice, raises concerns that avoidance of emulsifiers will considerably restrict food selection and negatively impact food‐related quality of life, but it is not based upon results of interventional studies that are directly applicable to the food intake in the community. This conundrum was approached by first attempting to define whether emulsifiers in the diet might have deleterious effects on the gastrointestinal tract of healthy adults. Contrary to effects anticipated from preclinical work, intestinal barrier function improved in an unstressed state associated with consuming a HED compared with a LED for 4 weeks [16]. In contrast, under stressed conditions, HED was associated with an exaggerated increase in small intestinal permeability while the LED provided durable protection from this effect [16]. Such unexpected observations led us to evaluate the effect of such diets on patients with active Crohn's disease. The lack of harmful signals from the present study suggests that the presence of an injured/inflamed intestine does not set up conditions for emulsifier‐damaging effects, as discussed above. Another high‐emulsifier diet that improves intestinal inflammation is exclusive enteral nutrition, a completely liquid diet that induces mucosal healing in active Crohn's disease [49]. Enteral formulas are ultra‐processed foods and contain a wide variety of emulsifiers [50]. Certainly, such observations together with the current data give reassurance that emulsifiers provided in the context of real‐world diets based on healthy eating guidelines over 4 weeks are unlikely to worsen disease activity and, conversely, that avoiding emulsifiers favourably affects disease activity. Such observations should lead to further critical examination of emulsifier effects in humans. Evaluation of longer duration is needed, and from a mechanistic point of view, the interaction between emulsifiers and other food complexes, their digestion and interaction with the gut microbiome in humans warrants investigation, so that the basis of dietary advice has more relevance.

Our study has a number of strengths. First, it was a double‐blinded randomised trial, where the participants, gastroenterologists who completed the ultrasound, laboratory staff and study coordinators were blinded to the randomisation. Second, it was a feeding study, and the dietary intervention and adherence were, therefore, highly controlled and monitored. Third, the primary outcomes were sensitive and objective measures of disease improvement.

However, the study had its limitations. First, it was limited to 4 weeks intervention, which was due to pragmatic and feasibility considerations, such as the financial cost and concerns that the risk of withdrawal from a study by participants who are eating a 7‐day meal plan for 4 weeks requires strict adherence. While the effects of high or low intake of emulsifiers might require a longer duration of exposure to emerge, the effects in preclinical work have occurred within a relatively short time frame. Second, as an exploratory study, the study cohort was small and, hence, underpowered to detect subtle changes or to avoid errors from multiple comparisons. However, post hoc examination of the likelihood of detecting differences in the major end points between diets that were rich or minimal in emulsifiers indicated that it was futile to continue with the same study design where most food was provided. Third, it included only adult patients with ileal involvement, so it is unclear if this is generalisable to other disease phenotypes. Fourth, the diets were based on the Australian Guide to Healthy Eating and comprised food from the Australian food supply, so again, translation into other countries may be difficult. Fifth, it is possible that emulsifiers have deleterious effects in the context of unhealthy diets, again limiting generalisation of the findings. Finally, there was no a priori plan to formally examine diet quality, specifically the comparison of the habitual diet with that of the interventional diets that were modelled on healthy diet guidelines, since the objective improvement in disease activity was not anticipated with both interventional dietary arms. Interpreting the dietary quality in the current small cohort by retrospectively applying one of over 20 different assessment tools developed to measure different elements of perceived dietary quality, as has been performed in epidemiological studies, would be challenging and it would be difficult to know what is clinically meaningful [51]. It is important to stress that both interventional diets were based on healthy eating guidelines, so any potential dietary quality confounders are minimal between the HED and LED as they were modelled on the same food group structure to meet nutritional requirements. Future assessment of these diets should incorporate validated dietary quality scores to enable better interpretation of habitual compared with interventional diets.

In conclusion, the content of emulsifiers in the context of diets that followed healthy eating guidelines had no apparent influence over intestinal inflammation in patients with mildly active Crohn's disease with ileal involvement over 4 weeks in this preliminary evaluation. In fact, significant improvement in intestinal inflammation measured sonographically and in rates of clinical remission, as well as quality of life, fatigue and central adiposity were observed for both dietary interventions. The mechanisms for the beneficial effects observed are not yet known. These findings suggest that restriction of emulsifiers as found in the food supply, when consumed as part of a healthy diet, may not influence clinical outcomes, at least over 4 weeks. Advice that foods containing emulsifiers in general should be avoided in patients with active Crohn's disease, as stated in dietary guidance, is not supported by the findings of this exploratory study.

## Author Contributions

**Jessica A. Fitzpatrick:** conceptualization, methodology, data curation, investigation, validation, formal analysis, writing – original draft, writing – review and editing. **Peter R. Gibson:** conceptualization, methodology, validation, formal analysis, supervision, writing – review and editing. **Kirstin M. Taylor:** methodology, resources, writing – review and editing. **Ellen J. Anderson:** writing – review and editing, methodology. **Antony B. Friedman:** methodology, writing – review and editing. **Zaid S. Ardalan:** methodology, writing – review and editing. **Rebecca L. Smith:** methodology, writing – review and editing. **Emma P. Halmos:** conceptualization, methodology, data curation, investigation, formal analysis, validation, supervision, funding acquisition, writing – review and editing.

## Conflicts of Interest

JAF: speaker honoraria Mindset Health Pty Ltd. and Pepsi Co. PRG: consultant or advisory board member for Anatara, Atmo Biosciences, Topas and Comvita; research grants for investigator‐driven studies from Atmo Biosciences and Mindset Health, and speaker honoraria from Dr. Falk Pharma and Mindset Health Pty Ltd; shareholder in Atmo Biosciences. KMT: Nil. EJA: Nil. ABF: shareholder in Atmo Biosciences. ZA: Nil. RLS: speaker fees/honoraria from Abbvie and Johnson & Johnson. EPH: received research grants from Mindset Health Pty Ltd and the Gastroenterological Society of Australia IBD Clinical Project award. She has received honoraria or consulted for Ferring, Janssen, Abbvie, Takeda, Shire, Sandoz and Dr Falk Pharma.

## Supporting information


Figure S1.

## Data Availability

Deidentified data will be shared on application and consideration by the authors.
