# Supplementary material for: Clinical Trial: The Effects of Emulsifiers in the Food Supply on Disease Activity in Crohn's Disease: An Exploratory Double‐Blinded Randomised Feeding Trial
Source: Aliment Pharmacol Ther. 2025 Feb 18;61(8):1276–89. doi: 10.1111/apt.70041 (PMC11950802; doi:10.1111/apt.70041)
Supplement: Supplementary file 1 — Figure S1. [file APT-61-1276-s001.docx]

**Supplementary Figure 1**. Seven-day meal plan of provided food (with the exception of fresh fruit and salad) on a high emulsifier diet (pink) and a low emulsifier diet (blue).


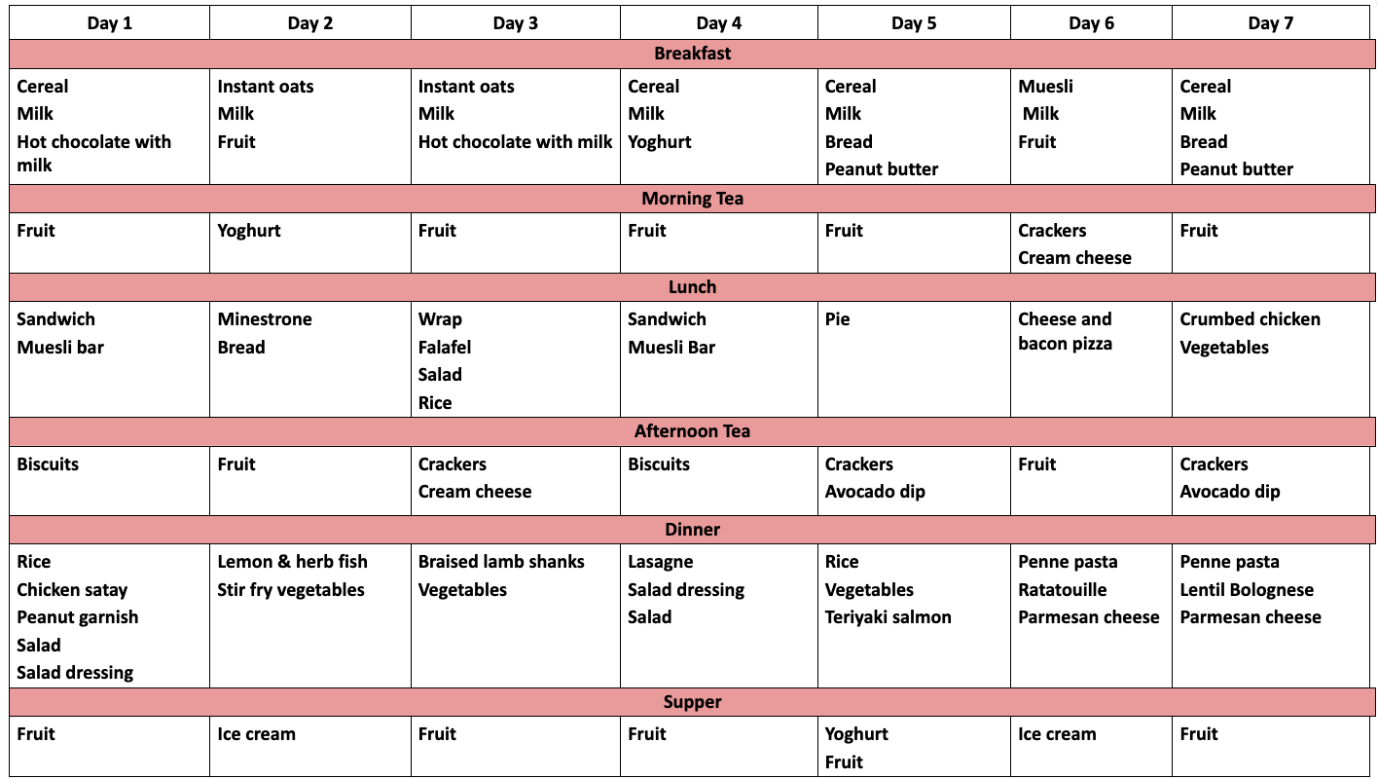
***High emulsifier diet***


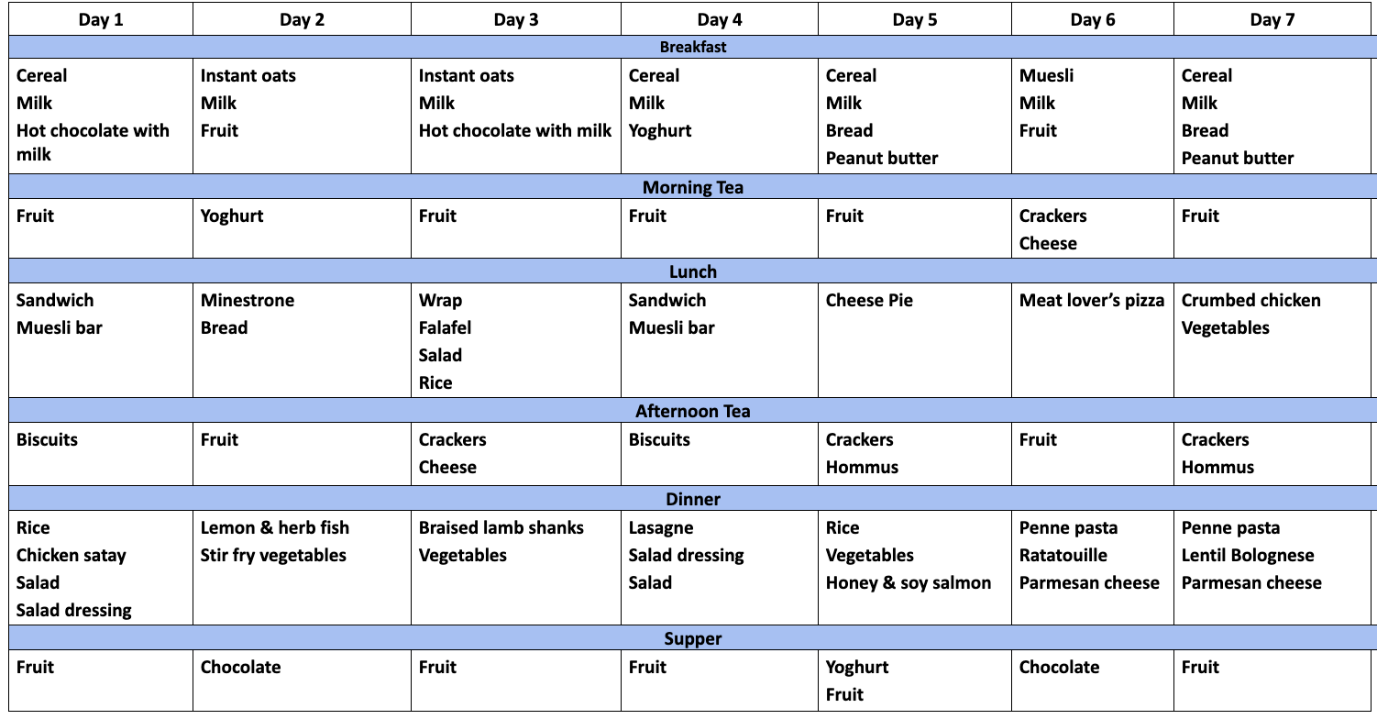
***Low emulsifier diet***
